# Glomus Tumor of the Lower Extremity Previously Misdiagnosed as Complex Regional Pain Syndrome in Close Proximity to a Myxofibrosarcoma: A Case Report

**DOI:** 10.5435/JAAOSGlobal-D-21-00311

**Published:** 2022-07-06

**Authors:** Alireza K. Nazemi, John Grossi, Felix B. Tavernier, Brendan F. Boyce, David E. Komatsu, Fazel A. Khan

**Affiliations:** From the Department of Orthopaedic Surgery, Stony Brook University Hospital, Stony Brook, NY(Dr. Nazemi, Dr. Komatsu, and Dr. Khan); the Marywood University, Scranton, PA(Grossi); and the Department of Pathology, Stony Brook University Hospital, Stony Brook, NY (Dr. Tavernier and Dr. Boyce).

## Abstract

Complex regional pain syndrome (CRPS) is a potentially devastating condition that can result in severe psychological and social morbidity. It is a diagnosis of exclusion, and other pathologic entities must be ruled out first. Glomus tumors are exquisitely painful benign vascular tumors that are most common in the hand and are rarely found in the lower extremity. Here, we present a case of a patient who developed a focus of severe anterior knee pain and tenderness a few months after a car accident that had been misdiagnosed as CRPS for 15 years. She coincidentally developed a sarcoma of her ipsilateral leg distal to this site. Magnetic resonance imaging of the sarcoma included the area of knee pain where, interestingly, it identified a separate small soft-tissue mass. A glomus tumor was diagnosed histologically in a needle biopsy specimen from this mass, which was resected along with the sarcoma. For the first time in 15 years, despite the additional sarcoma surgery, she reported relief of her pain and complete resolution of her “CRPS.”

Complex regional pain syndrome (CRPS) is characterized by continuous localized pain that is disproportionate to any causative trauma or lesion. The pain is considered regional because it is not in an identifiable nerve distribution or dermatome, and it may consist of a constellation of abnormalities in sensation or motor function along with vasomotor, sudomotor, or trophic findings.^1,2^ CRPS is a diagnosis of exclusion. The Budapest diagnostic criteria comprise the most widely used system for diagnosing CRPS^1,3,4,5,6^ and emphasize, “There is no other diagnosis that better explains the symptoms.” Patients often present with skin-related changes, as well as severe and often debilitating pain. The diagnosis and treatment of CRPS may be challenging. Other maladies often considered in the differential diagnosis include infection, deep vein thrombosis, Raynaud disease, thoracic outlet syndrome, vascular insufficiency, and psychiatric diseases.^5,7,8^ However, the diagnostic pathways described in the literature often neglect to mention soft-tissue tumors.

Glomus tumors are rare, benign, vascular neoplasms that arise most commonly subungually in fingers. Although they occur almost exclusively in the upper extremity, with ∼75% in the hand,^9^ rare cases have been reported in the lower extremity.^10,11,12,13,14^ Glomus tumor characteristics include pain and tenderness at the location of the nodule and temperature sensitivity, especially to cold.^10,15^ They are commonly misdiagnosed and can cause persistent pain and discomfort if they are not treated effectively. In one series of glomus tumors from 1995 to 2009, the average time to diagnosis was 3.3 years.^9^ Surgical excision is the standard of care.^10^

This study describes a patient misdiagnosed with CRPS affecting the anterior knee region for 15 years. Magnetic resonance imaging (MRI) of the affected area had not been done. She coincidentally presented with a soft-tissue sarcoma distal to her area of knee pain; this prompted an MRI, which revealed, in addition to the soft-tissue sarcoma, a small mass in the area corresponding to her “CRPS.”

## Case Presentation

A 68-year-old woman was referred by a trauma surgeon for an orthopaedic oncology consultation about an expanding anterior left leg soft-tissue mass. She had returned to the trauma surgeon for a routine follow-up 1 year after cephalomedullary nailing of an ipsilateral intertrochanteric fracture. Her medical history included a distal pancreatectomy and splenectomy for pancreatic cancer 6 years previously with no known metastases. She was also diagnosed with diabetes, hypertension, hyperlipidemia, osteoporosis, and a transient ischemic attack. Her surgical history included resection of the carotid body and adrenal tumors 12 years before presentation, and workup for pheochromocytoma was negative. She had no endocrine or oncological concerns since then.

A diagnosis of CRPS of the left lower extremity was made after a motor vehicle accident 15 years before her recent presentation with the leg mass. After the motor vehicle accident, she had mild knee pain, but within 1 year, the pain had worsened, was severe, was burning in nature, and was accompanied by occasional bouts of severe allodynia, which prevented her from actively extending her knee. She had been seen by five neurologists, who had all diagnosed CRPS, and an outside orthopaedic surgeon who recommended physical therapy and pain control. Her pain was uncontrolled on oral pain medications, and she was unable to complete physical therapy. She was referred to pain management where she had four rounds of intravenous infusion therapy with lidocaine, ketamine, and midazolam. She was offered a left L3 sympathetic nerve block but declined it.

The orthopaedic oncologist observed a 5-cm ovoid, firm mass in her proximal anterolateral leg that was mildly tender to palpation. She had no associated skin changes and was neurovascularly intact distally. She resisted examination of the area of focal “CRPS” located proximal to the mass because it was extremely sensitive to touch but allowed palpation of the tissues around it, including the soft-tissue mass.

**Figure 1:**
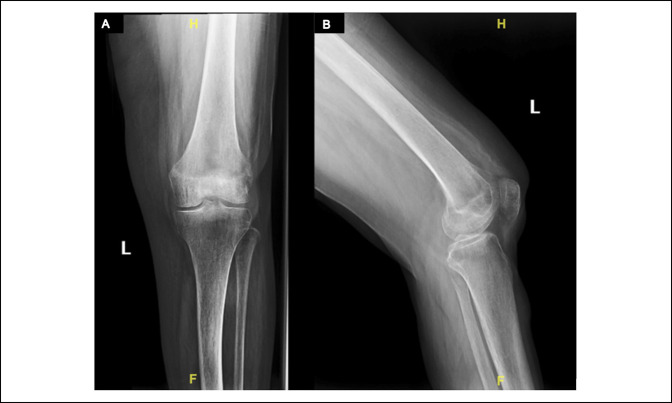
**A**, Anterior-posterior and (**B**) lateral radiographs of the left knee demonstrate no acute fracture or dislocation. No evidence of osteolytic or osteoblastic changes about the knee.

**Figure 2:**
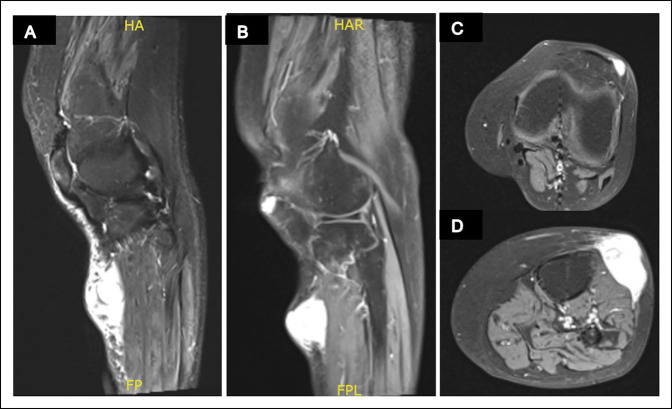
Radiographs showing MRI scans of the knee and proximal leg demonstrating both lesions. **A,** Sagittal T2 with hyperintensity indicative of the ovoid sarcomatous mass on the proximal anterolateral aspect of the leg. **B,** Sagittal fat-saturated T1 post-contrast demonstrating both the sarcomatous mass and a more proximal circumscribed hyperintensity about the anterolateral aspect of the patella which corresponded with the glomus tumor. Axial fat-saturated T1 post-contrast images (**C**) proximally at the level of the glomus tumor and (**D**) distal to the knee at the level of the soft-tissue sarcoma.

Radiographs of the left knee showed no acute fracture, dislocation, or destructive bony changes (Figure 1). An MRI scan with and without contrast of her left lower extremity showed a heterogenous solid mass within the subcutaneous tissues along the anterolateral calf (Figure 2), indeterminate but could cause concern for a soft-tissue sarcoma, and a small T2 hyperintense nodule within the subcutaneous tissues along the anterior aspect of the patella, which could represent a number of pathologies, including prepatellar bursitis, ganglion cyst, glomus tumor, or even potentially be a part of the sarcoma itself.

The larger mass was rapidly growing and could be a concern for malignancy, and an ultrasound-guided core needle biopsy of that mass was expedited and conducted at bedside without general anesthesia. However, the patient's hypersensitivity in the region of her previously presumed “CRPS” prevented biopsy under local anesthesia. Therefore, an ultrasound-guided core needle biopsy was conducted of the T2-hyperintense region under general anesthesia.

**Figure 3:**
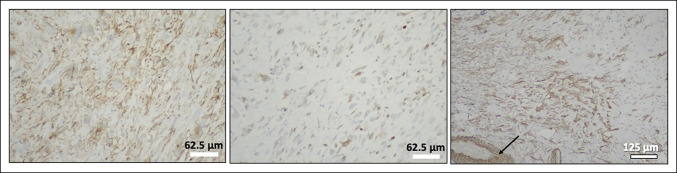
Images demonstrating immunohistochemistry of tumor cells showing focal membranous expression of CD99 (**A**), focal nuclear/cytoplasmic expression of BCL-2 (**B**), and focal cytoplasmic/membranous expression of smooth muscle actin (**C**), with vascular smooth muscle cells serving as an internal positive control (arrow).

Histologic examination of the biopsy specimen of the rapidly growing leg mass showed a spindle cell tumor with myxoid stroma, focal sclerosis, moderate nuclear pleomorphism, multinucleated giant cells, mast cells, and up to seven mitoses/10 high-power fields, with no tumor necrosis. Immunohistochemistry showed that the tumor cells had focal membranous expression of CD99, nuclear/cytoplasmic expression of B-cell lymphoma 2, and cytoplasmic/membranous expression of smooth muscle actin and CD34, consistent with an intermediate-grade spindle cell sarcoma (Figure 3). Additional immunohistochemical studies showed focal cytoplasmic expression of MUC4 and no expression of STAT6, consistent with a low-grade fibromyxoid sarcoma (Figure 3). A staging chest CT scan and whole-body positron emission tomography-CT scan showed no distant metastatic disease. The positron emission tomography-CT showed increased uptake in the regions of both leg masses suggestive of a malignant neoplasm.

**Figure 4:**
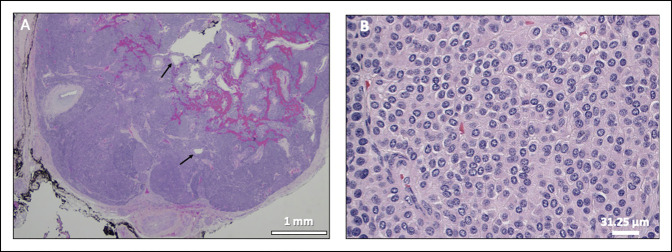
**A,** Image showing a circumscribed glomus tumor with a fibrous capsule, dilated vessels (arrows), hemorrhage, and nests/sheets of tumor cells separated by fibrous bands. **B,** Image showing uniform small round cells with distinct cell borders, centrally placed round-to-oval nuclei with relatively coarse chromatin, and granular eosinophilic cytoplasm. Hematoxylin and eosin staining.

**Figure 5:**
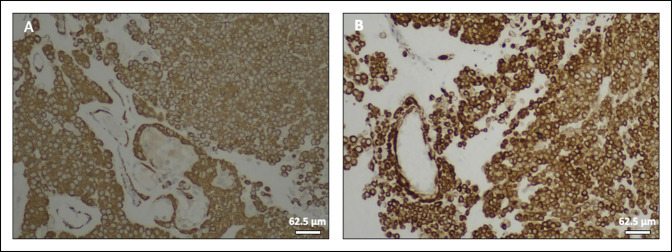
Images demonstrating immunohistochemistry of glomus tumor cells showing strong cytoplasmic expression of smooth muscle actin (**A**) and calponin (**B**).

Histologic sections of the biopsy from the previously diagnosed “CRPS” showed capillaries surrounded by a nested and cord arrangement of small round cells with distinct cell borders, central round-to-oval nuclei with relatively coarse chromatin, and granular eosinophilic cytoplasm, which strongly expressed smooth muscle actin, calponin, and vimentin, with no expression of S100, chromogranin, synaptophysin, or pancytokeratin, consistent with glomus tumor (Figures 4 and 5).

Interestingly, after the glomus tumor core needle biopsy, the patient was almost pain-free at the previously hypersensitive region in her anterior knee for the first time in 15 years, essentially ruling out her diagnosis of CRPS. She now allowed palpation of the anterior knee region, presumably because the biopsy had decompressed/partially removed the glomus tumor. Perhaps, owing to the small size of the lesion, a notable portion of the pathology was removed by the core needle biopsy. Therefore, the pain relief that would typically be expected after surgical excision was afforded after the biopsy.

After the biopsy results were analyzed, the patient was admitted for wide resection of the sarcoma and marginal resection of the glomus tumor simultaneously. A longitudinal incision was made over the site of the previous glomus tumor biopsy; a small round mass was identified in the subcutaneous fat and this mass was resected *en bloc*. The mass appeared bluish and was consistent with the classic appearance of a glomus tumor, and no residual abnormal tissue was visualized. The larger mass was then excised *en bloc* through a large ellipsoid incision that included the sarcoma biopsy site. The sarcoma excision included a strip of the underlying periosteum and surrounding musculature because of MRI findings of a questionable tail of the mass extending to the tibial crest. Tumor margins were confirmed to be negative on gross examination intraoperatively by a pathologist (B.F.B.). The patient did well postoperatively and remained hospitalized until reconstructive surgery using a medial gastrocnemius rotational flap was conducted for the sarcoma excision 1 week later, to allow for a complete histologic analysis of surgical margins.

**Figure 6:**
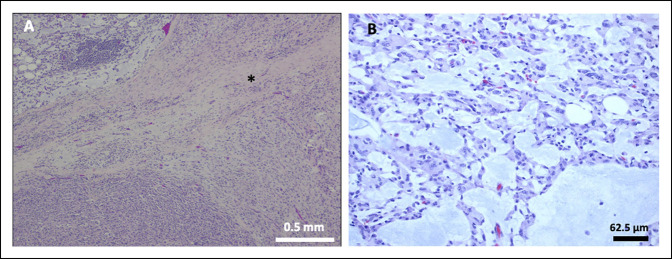
**A,** Image showing myxofibrosarcoma with dense fibrous bands (asterisk) and a heterogenous appearance, including myxoid (top left) and solid (bottom left) areas. **B,** Image showing the myxoid area of the tumor with epithelioid tumor cells. Hematoxylin and eosin staining.

**Figure 7:**
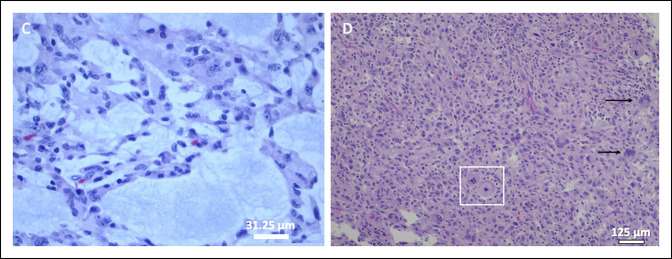
**A,** Image showing epithelioid tumor cells in a myxoid background. **B,** Image showing medium-to-large tumor cells with epithelioid features, marked nuclear pleomorphism, mitoses (inside box), and multinucleation (arrows). Hematoxylin and eosin staining.

The sarcoma specimen comprised a 10.5 × 7.0 cm skin ellipse with attached subcutaneous tissue and muscle, which contained a white-tan 4.0 × 3.5 × 3.3 cm centrally located nodule bulging under the skin surface. Serial sectioning revealed that the lesion was well circumscribed and multinodular, with pink-tan and yellow-tan cut surfaces. Histologic analysis showed tumor cells with spindle, round, and epithelioid features; focally marked nuclear pleomorphism; and multinucleated tumor giant cells in a myxoid background, with dense fibrous bands separating myxoid and solid regions, up to 21 mitoses/10 high-power fields, but no necrosis (Figures 6 and 7). Immunohistochemistry showed that the tumor cells were focally positive for CD99 and BCL-2 and negative for CD34, S100, desmin, and myogenin. Immunostaining for MDM2 and CDK4, markers used to distinguish dedifferentiated liposarcomas from poorly differentiated sarcomas, and MUC4 was negative.^16^ A diagnosis of high-grade myxofibrosarcoma with focally epithelioid features was rendered.

The patient was given postoperative radiation therapy for 6 weeks, and a repeat MRI scan showed no residual sarcoma. She is doing well at more than 2 years postoperatively with no evidence of disease on surveillance MRI scans of the knee and CT scans of the chest. She remains free of leg pain.

## Discussion

Glomus tumors are uncommon and, in one study, comprised only 1.6% of 500 consecutive soft-tissue tumors.^17^ Although typical glomus tumors share the triad of pain, point tenderness, and cold hypersensitivity,^18^ extradigital glomus tumors are rarer and often do not share this constellation of symptoms.^19,20^ Extradigital glomus tumors can appear in the upper and lower extremities and multiple other sites in the body.^21^ Owing to their unusual nature and nonuniform symptoms, they tend to present with a longer duration of time to diagnosis. Lower extremity extradigital tumor case reports have involved patients with burning pain, difficulty with ambulation, and even unilateral limb atrophy.^22^ Often, extradigital glomus tumors are not diagnosed until an MRI is done. In several reported cases of lower extremity glomus tumor, MRI of the painful regions was often done several months to years after the patient began experiencing pain.^19-21^ Unfortunately, the patient in this case experienced a delay in diagnosis as well.

MRI is a useful tool in the diagnosis of glomus tumor. Findings of glomus tumors on MRI are often consistent with homogenous well-circumscribed lesions with hypointensity on T1-weighted images and hyperintensity on T2-weighted images along with contrast enhancement.^23^ MRI has been shown to have a high positive predictive value for glomus tumors as small as 2 mm in diameter.^24^

Although glomus tumors are rare, they can cause symptoms, which were misdiagnosed as CRPS in the upper extremity in two previously reported cases.^25,26^ As in this case, these patients noticed a significant decrease in symptoms following excision of the tumors. Glomus tumors are rare in the lower extremity, which might account for the repeated misdiagnosis of CRPS by an interdisciplinary team of physicians in this case. In addition, a glomus tumor near a soft-tissue sarcoma in the lower extremity has not been reported previously.

Although in this case, the orthopaedic oncologist opted to first have the painful soft-tissue mass biopsied before excision, a primary excisional biopsy could have been justified. The history was suggestive of glomus tumor, the mass was small and superficial enough to address safely through an excisional biopsy, and the core needle biopsy itself was not trivial because it required general anesthesia because of the patient's hypersensitivity. However, the senior author (F.A.K.) elected for a core needle biopsy because of the highly unusual nature of this case. Several years of misdiagnosis by multiple physicians and the coincidental fact that the patient developed a large soft-tissue sarcoma near the undiagnosed soft-tissue mass raised questions about the mass representing something other than a glomus tumor and the possibility of a relationship to the nearby malignant mass. Core needle biopsy has been shown to provide high diagnostic accuracy for soft-tissue masses^27^ and, in this case, was used to provide a tissue diagnosis before initiating treatment. General anesthesia was necessitated because the patient had exquisite local hypersensitivity to the extent that even contact with clothing could cause pain in the region of her anterolateral leg.

It is important to appreciate that CRPS is a diagnosis of exclusion. On plain radiography, CRPS has been associated with periarticular osteopenia and patchy osteoporosis.^28^ Three-phase bone scanning has been reported to have a high negative predictive value to rule out CRPS type I,^3,29,30^ which presents without an identifiable nerve lesion and accounts for approximately 80% to 90% of CRPS cases.^2,4^ In a three-phase bone scan, abnormal flow patterns and vasomotor instability are seen in the first and second phases while increased periarticular uptake is seen in the third phase.^3,30^ CRPS type II, which presents with an identifiable nerve lesion, may show a decrease in nerve conduction velocity and increased sensorimotor latency on electromyography.^31^ Other diagnostic tools for CRPS include bone densitometry, infrared thermography, sweat testing, sympathetic blocks, bedside clinical examination methods, and targeted questionnaires.^1,28,32^

MRI has been largely excluded in the CRPS literature because it has been shown to have lower sensitivity and specificity than a three-phase bone scan for CRPS.^33^ Owing to nonuniform MRI findings, such as an absence of bone edema up to 50% of the time, MRI has been viewed as only an adjunct to help rule out alternative diagnoses.^34^ Overall, most of the research on MRI and CRPS is regarding brain MRIs and analyzing the neurologic involvement of this poorly understood condition.^35,36^ Nonetheless, MRI is useful in diagnosing pathologic entities, such as tumor or infection. Although a tumor may be unlikely to cause symptoms mimicking CRPS, it did in this case. In addition, advanced imaging was included in the workup only after the development of a visible palpable mass close by the painful region. Knowledge of CRPS-like symptoms as a possible presentation of glomus tumor and a thorough clinical workup, including advanced imaging, could result in a prompter diagnosis, reduce the burden of pain, and facilitate appropriate treatment.
